# Use of a matchline dosimetry analysis tool (MDAT) to quantify dose homogeneity in the region between abutting tangential and supraclavicular radiation fields

**DOI:** 10.1120/jacmp.v11i4.3294

**Published:** 2010-07-27

**Authors:** Kenneth L. Homann, B. Earl Gates, Mohammad Salehpour, David S. Followill, Steven M. Kirsner, R. Allen White, Thomas A. Buchholz, Karl L. Prado

**Affiliations:** ^1^ Department of Imaging Physics The University of Texas M. D. Anderson Cancer Center Houston TX; ^2^ OncoLogics, Inc. Cancer Center Lafayette LA; ^3^ Department of Radiation Physics The University of Texas M. D. Anderson Cancer Center Houston TX; ^4^ Department of Biostatistics and Applied Mathematics The University of Texas M. D. Anderson Cancer Center Houston TX; ^5^ Department of Radiation Oncology The University of Texas M. D. Anderson Cancer Center Houston TX; ^6^ Department of Radiation Oncology The University of Maryland School of Medicine Baltimore MD USA

**Keywords:** field abutment, matchline dose, film dosimetry, multileaf collimators, whole breast irradiation

## Abstract

In this work, we develop and test a matchline dosimetry analysis tool (MDAT) to examine the dose distribution within the abutment region of two or more adjoining radiotherapy fields that employ different blocking mechanisms and geometries in forming a match. This objective and quantitative tool uses calibrated radiographic film to measure the dose in the abutment region, and uses a frequency distribution of area versus dose (a dose‐area histogram) to visualize the spatial dose distribution. We tested the MDAT's clinical applicability and parameters by evaluating the dose between adjacent photon fields incident on a flat phantom using field‐matching techniques employing collimator‐jaw and multileaf collimator (MLC) configurations. Additionally, we evaluated the dose in the abutment regions of four different clinical tangential‐breast and supraclavicular matching techniques using various combinations of collimator and MLC matches. Using the MDAT tool, it was determined that a 1 cm abutment region width (centered about the theoretical matchline between fields) is the most appropriate width to determine dose homogeneity in a field matching region. Using the MDAT, both subtle and large differences were seen between fields that used MLCs to form the match, compared to flat edge devices such as collimators and external cerrobend blocks. We conclude that the MDAT facilitates a more precise evaluation of the distribution of dose within the region of abutment of radiotherapy fields.

PACS number: 87.55.dk

## I. INTRODUCTION

In radiation therapy, clinical situations often arise where the “target volume” is greater than the volume that can be encompassed by a single set of treatment fields. This occurs especially during head and neck, breast, and craniospinal irradiation.^(^ ^1^ ^–^ ^3^ ^)^ In these instances, multiple treatment fields are abutted or matched to ensure full dose coverage of the target volume. The fundamental goal of field matching is to produce a dose within the abutment region that is equivalent to the dose within the abutting fields.

The process of field abutment entails matching the edges of the treatment fields in such a manner as to create a seamless transition both dosimetrically (homogenous dose distributions) and geometrically (nondivergent beams) from one field to the next, thus forming a “perfect match”. In craniospinal irradiation, where parallel superior and inferior spine fields are abutted at depth, the edges of the fields produce a match along a single line (i.e., a matchline).^(^ ^1^ ^)^ However, in breast and in head and neck irradiation, where anterior supraclavicular fields and tangential breast fields or lateral head and neck fields are abutted, respectively, the fields are matched in such a way that a ‘match plane’ is produced rather than a simple matchline.^(^ ^2^ ^–^ ^3^ ^)^ The edges of the matched fields can be produced by collimator jaws, by customized blocking, or by multileaf collimators (MLCs).^(^ ^2^ ^–^ ^4^ ^)^ Regardless of the field‐matching technique used, the implicit requirement is that the dose in the abutment region be known, and that it ideally is comparable to – if not equal to – the dose delivered by the matched fields.

Of particular interest to us recently was the dose uniformity between supraclavicular and tangential fields used to treat the intact breast. While IMRT breast treatment may have become more widespread,^(^ ^5^ ^–^ ^6^ ^)^ possibly eliminating matchline dosimetry concerns, many institutions, including ours, still apply a three field breast forward planned technique for intact breast treatments. In order to form our matchline between the tangents and supraclavicular field, we employ the “Rod and Chain” technique.^(^ ^2^ ^)^ In this technique, an external block is used to shape the superior border of the tangential fields in order to achieve a geometric match with the inferior border of the anterior supraclavicular (SC) field. The use of these blocks became impractical, however, when we began treating our breast patients on treatment units with tertiary multileaf collimators because of the reduced clearance between the patient and the block tray. Previously, we had treated breast patients on a unit in which the multileaf collimator replaced the lower jaw. Thus, a principal motivation of this project was development of an objective means of evaluating dose between these adjacent fields.

In the present work, we describe a film‐based matchline dosimetry analysis tool (MDAT) that we developed to quantitatively compare field‐matching techniques. We characterized our film dosimetry system and compared our results with published data.^(^ ^6^ ^–^ ^9^ ^)^ After using this system to measure the dose in each treatment field, we used the MDAT in a flat phantom geometry to assess the dose distribution within the abutment region between fields that were shaped using three jaw and MLC configurations. We then used the validated tool to assess the dose in the abutment regions between tangential breast and SC fields produced, using four different field‐abutment techniques. The MDAT has allowed us to more precisely and objectively describe the dose distribution in the region of abutment of radiotherapy fields.

## II. MATERIALS AND METHODS

### A. Film dosimetry system

Dose was measured using Kodak EDR2 radiographic film (Kodak Scientific Imaging, Rochester, NY). Film was developed on a Kodak RP X‐OMAT Processor (model M6B; Eastman Kodak Company, Rochester, NY), digitized on a Vidar VXR‐16 Dosimetry Pro 16‐bit scanner (Vidar Systems Corporation, Herndon, VA), and analyzed using two software programs that were developed in‐house at The University of Texas M. D. Anderson Cancer Center. The first program was DoseLab, which consists of MATLAB (The Math Works, Inc., Natick, MA) code designed to convert film optical density to a pixel matrix of absorbed‐dose values; the second program was the MDAT, which consists of a Microsoft Excel spreadsheet that “bins” the absorbed‐dose pixels within a field abutment region into discrete dose ranges, and then presents them in a histogram distribution. To eliminate dosimetric changes that may result from daily variations in film processing, an optical density‐to‐dose calibration film was run every time film was used, immediately preceding experimental film.^(^ ^10^ ^–^ ^11^ ^)^ We used a single film calibration (SFC) technique to acquire the film's sensitometric curve.^(^ ^10^ ^)^ In this film irradiation technique, a single film is irradiated to nine dose levels using a series of small fields (3×3 cm) produced by a step‐and‐shoot intensity‐modulated radiotherapy sequence.

The calibrated film dosimetry system was then validated under conditions of known dose distributions. Crossplane (XP) and percent depth dose (PDD) profiles were measured in RMI Plastic Water (Gammex‐RMI, Inc., Middleton, WI.) with the film dosimetry system, and then compared to ion chamber measurements made in water with a Scanditronix‐Wellhofer 3D Relative Dosimetry System (IBA Dosimetry America, Bartlett, TN.). The ion chamber used was a 0.04‐cc Wellhofer CC04 chamber. We used 6 MV X‐ray beams of three field sizes: $5\times 5{\text{cm}}^{2},10\times 10\;{\text{cm}}^{2}$, and $20\times 20\;{\text{cm}}^{2}$. XP profiles were obtained at depths of $d_{\max}$, 5 cm, 10 cm, and 15 cm. A total of 5 films (XP films + PPD films) for each field size were obtained. Repeat films were used to determine the reproducibility (average standard error) of the film dosimetry system. Three films were irradiated for each field size‐and‐depth combination, yielding a total of 45 films for analysis. The ion chamber and average film PDD and XP profiles were normalized, co‐registered in space, and then compared.

### B. Analysis of measured dose in the abutment region: the matchline dosimetry analysis tool (MDAT)

Although a single dose profile can be useful for relative dose characterization (i.e., it provides spatial dose information), it has some limitations. A single dose profile may not adequately characterize matchline dosimetry. The dose within an abutment region (area or volume), not within a line, is of greater interest and relevance in dosimetry analysis than is relative dose along a single line. In addition, a single dose profile could offer minimal insight into the dose homogeneity within the matching region, since there is a large degree of uncertainty involved in identifying the profile's location relative to the theoretical matchline between the two fields. This uncertainty may become larger when comparing matchline profiles between multiple pieces of film.

To overcome these spatial uncertainties and minimal dose information, and to develop a more comprehensive method of characterizing dose homogeneity, we used multiple adjacent and parallel profiles centered about the central theoretical matchline to evaluate the dose within the field abutment region. By doing so, we formed an area centered about the theoretical matchline rather than a single dose profile. The spatial and relative dose information of the pixels within the match region can then be presented in an area‐frequency distribution in a form analogous to the commonly used dose‐volume histogram (DVH). Because this planar frequency distribution shows the relationship between dose and area on film, it is called a dose‐area histogram (DAH).^(^ ^12^ ^)^

**Figure 1:**
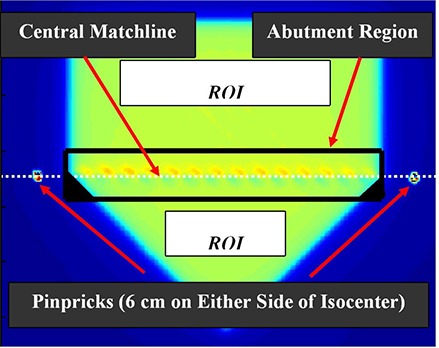
Schematic representation of the experimental setup used to determine the position of the central matchline and the location of the abutment region. The pinpricks located outside of the field (6 cm on either side of the isocenter) are placed on the central matchline. The abutment region encompasses an equal area extending superiorly and inferiorly relative to the central matchline. The regions of interest (ROI) are used to determine the dose that will be used to normalize the pixels' dose values within the abutment region.

The DAHs that form the basis of the MDAT were normalized using the same method that DVHs are normalized. The area axis (y‐axis or ordinate) is normalized to the total area of the match region which is equal to the match region length times the abutment region width. The dose axis (x‐axis or abscissa) is normalized to the average dose within the two abutting fields. The latter normalization is accomplished by establishing regions of interest (ROIs) within each treatment field at the depth at which abutment dosimetry is being evaluated, and then computing the average doses within the two ROIs (Fig. 1). Thus, the abutment region dose magnitude and homogeneity can be evaluated relative to the dose existing within the two abutting fields.

### C. Determination of width and resolution of the field abutment region

The most suitable abutment region width was determined empirically. Multiple abutment region widths, ranging from 0.03 cm to 1.75 cm centered about the theoretical matchline, were evaluated using differential DAHs. Because a single pixel in the DoseLab software has a size of approximately 0.03 cm, the abutment region widths evaluated contained the number of profiles shown in Table 1.

**Table 1:** Abutment region widths in cm, versus the numbers of profiles contained in each region. Shown for each width are the total numbers of single parallel and adjacent profiles existing in the region of the match, and the number of profiles located on each side of the central profile.

*Abutment Region (width (cm))*	*Total Number of Profiles in the Match Region*	*Number on Each Side of Central (theoretical) Match*
0.03	1	0
0.10	3	1
0.25	7	3
0.50	15	7
0.75	21	10
1.00	29	14
1.25	35	17
1.50	43	21
1.75	49	24

We also determined the resolution of the dose bins of the differential DAHs that were used to evaluate the abutment regions widths. In general, the bin sizes of histograms are selected based on the errors inherent in the experimental system.^(^ ^12^ ^)^ In our study, the experimental error was evaluated when our film dosimetry system was characterized. Nevertheless, to examine the effect of bin size on the MDAT, we computed integral MDATs of the jaw–MLC–0° abutment region, using four different bin sizes that ranged from 0.5% relative dose to 5.0% relative dose and then compared them.

### D. Initial evaluation of dose within an abutment region: flat phantom

To form an abutment region between two fields, we adjoined three pairs of anteroposterior fields, with each field in a given pair sharing the same isocenter. The fields were produced on a Varian 2100C accelerator equipped with a Millenium MLC (Varian Medical Systems, Milpitas, California). A “superior field” was half‐beam blocked using an asymmetric jaw, producing a field 5 cm in length and 10 cm in width. The latter dimension represents the length of the field's inferior border, to which the second field was abutted. The dimensions of the superior field were kept constant throughout the entire study.

**Figure 2:**
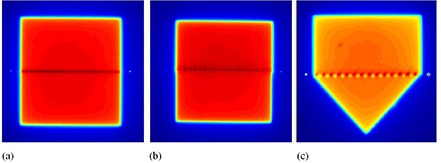
Screen captures of Kodak EDR2 film‐density images depicting the three matching techniques: (a) Jaw–Jaw, (b) Jaw–MLC–0°, and (c) Jaw–MLC–45°. Note the effects of the MLC on the abutment region in (b) and (c), compared to the straight collimator edge in (a).

Three “inferior fields” were developed to match to the inferior border of the constant superior field. The first consisted of a field that mirrored the superior field (i.e., this first inferior field was a beam‐split field ($5\times 10\;{\text{cm}}^{2}$) in which the 10 cm superior border was formed with an asymmetric jaw). The field abutment or match produced by this first pair of abutted fields was called the “Jaw–Jaw” match (Fig. 2(a)). The second field also consisted of a $5\times 10\;{\text{cm}}^{2}$ beam‐split field but, in this case, the MLC leaves, rather than an asymmetric jaw, were used to produce the split beam and the match with the superior field's inferior border. In this configuration, the MLC leaves were positioned perpendicularly to the field edge that they formed. The match produced by this second pair of fields was called the “Jaw–MLC–0°” match (Fig. 2(b)). The third field consisted of a beam‐split ($5\times 10\;{\text{cm}}^{2}$) field but, in this case, the MLC was rotated 45° and its leaves were again positioned to form a common border with the superior field's inferior border. The match produced by this field orientation was called the “Jaw–MLC–45°” match (Fig. 2(c)). It should be noted at this point that the MLC leaves were positioned using the “cross‐bound” mode of MLC‐leaf placement. This is defined in the AAPM Task Group 50 report as the “leaf position [that] is selected such that the treatment field contour bisects the projection of the leaf end”.^(^ ^13^ ^)^

Films were irradiated in plastic water at 95 cm source‐to‐surface distance using accelerator monitor unit settings that were adjusted to deliver equal doses to the film from both the superior and inferior fields. The films were placed at 2 cm and 5 cm depths, centered on and perpendicular to the common central axes of the beams. The location of the “theoretical” matchline between the fields was marked on each film by piercing the film with a sharp point ~1 cm beyond the field abutment region (Fig. 1). This theoretical matchline represented the center of the abutment region that is defined below.

After selecting a suitable abutment region width and MDAT dose bin size, we evaluated the dose between the adjoining flat phantom fields described in Section II.B. As mentioned above, each experiment was repeated three times and the results were averaged. Since dose values are binned in the MDAT, the average MDAT was obtained by averaging the areas contained in each corresponding dose bin for each film.

### E. Characterization of the dose in abutment regions: clinical breast fields

As a final test of the MDAT, the dose between clinical tangential breast and supraclavicular fields was evaluated using a modified axially‐sliced anthropomorphic phantom. By using an actual breast patient's CT dataset as a basis, the phantom (fabricated by The Phantom Laboratory, Salem, N.Y.) was custom‐made to mimic as close as possible a typical breast cancer patient.

As stated above, the anthropomorphic phantom was sliced axially, as is standard for most phantoms produced commercially. This presents a problem for this body of work as all previous measurements have been done in the coronal plane. To resolve this issue, a coronally‐sliced insert of an anthropomorphic phantom was manufactured. This insert involves the anatomical region containing the abutment region (RANDO Phantom slices #9‐13). The phantom was scanned on a Philips AcQSim CT scanner (Philips Medical Systems, N.A., Bothell, WA) and then planned on Pinnacle^3^ treatment planning system (Philips Medical Systems, N.A., Bothell, WA).

**Figure 3:**
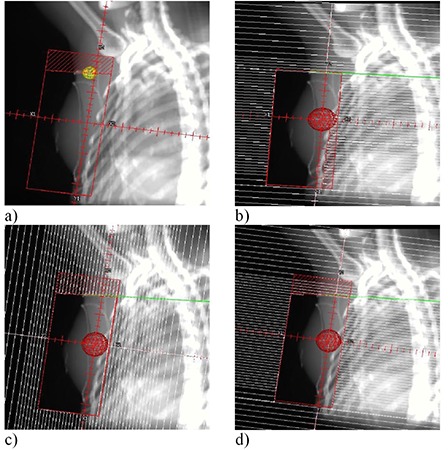
Screen captures from Pinnacle^3^ TPS depicting the four matching configurations tested for the clinical breast fields: a)External Block – an external cerrobend block is placed on the tangent fields to form the match between these fields and the anterior supraclavicular field; b) Jaw–Jaw – the collimator jaw now forms the match and MLC leaves are used to block the lung fields (Note how this is the only instance where the MLC is used to block the lung; this is required to compensate for the portion of lung that is unshielded in order to use the rotation of the collimator to form the “perfect” match); c)MLC‐Perpendicular – the leaves are brought in a closely approximated “perpendicular” orientation relative to the matchline; d) MLC‐Parallel – leaves are brought in a closely approximated “parallel” orientation relative to the matchline. Notice the orientation of the leaves for b), c) and d). Additionally, recall that the MLC‐Parallel technique is used for < 10 % of the MLC‐Perpendicular treatment in order to orient the leaves to perform “field‐in‐field” reduction of hot spots in the tangent fields.

Typical planning parameters, in terms of field coverage and geometry, were used. The junction between the tangential and SC fields was formed using four different methods of defining the superior border of the tangential fields: a smooth custom block (External Block) (Fig. 3(a)), a collimator jaw (Jaw‐Jaw) (Fig. 3(b)), an MLC (Fig. 3(c)) oriented such that its leaves were oriented essentially perpendicular to the junction (MLC‐Perpendicular), and an MLC (Fig. 3(d)) oriented such that its leaves were oriented roughly parallel to the junction (MLC‐Parallel). For the MLC‐Perpendicular technique (Fig. 3(c)), a small portion of the treatment (< 10% total dose) was conducted with the MLC‐Parallel configuration in order to orient the leaves in such a manner as to be able to use the “field‐in‐field” dose‐compensation technique^(^ ^6^ ^)^ that we employ to reduce hot spots within the tangents.

**Figure 4:**
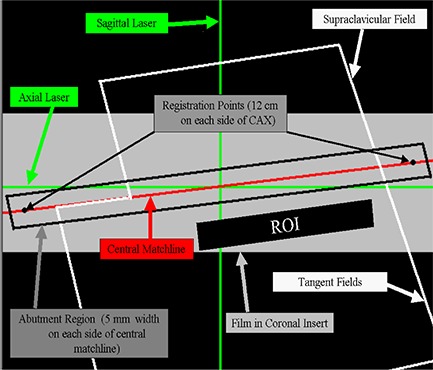
Cartoon depicting the clinical breast field setup and evaluation methods, highlighting the coronal plane at a depth of 3.5 cm.

Within each field‐matching configuration, the doses within the junction between the tangential and supraclavicular fields were evaluated in a coronal plane in the phantom at a depth of 3.5 cm from the anterior surface at the level of the junction. The MDAT was used in the anthropomorphic phantom in the same fashion as it was in the flat phantom measurements, with one exception. With the anthropomorphic phantom, the ROI used for normalization was located within the tangent fields only, immediately inferior to the abutment region. The tangent fields alone were used to determine the normalized dose since the tangent fields are designed to deliver a uniform prescribed volumetric dose to the entire field (i.e., 2.0 Gy). Conversely, the SC field has a decreasing dose as depth increases (Fig. 4). Each of the junction configurations was evaluated in this fashion three separate and distinct times, and the results for each were averaged as previously described.

## III. RESULTS

### A. Film dosimetry system

**Figure 5:**
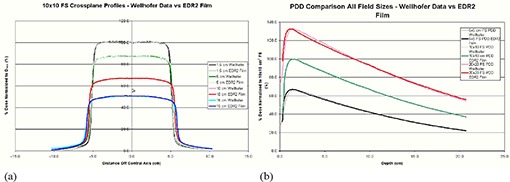
Crossplane (XP) profiles (a) and percent depth doses (PDDs) (b) obtained using the Wellhofer CC04 ion chamber in water and the Kodak EDR2 film in plastic water.

We tested and verified the film dosimetry system before using it to analyze the three matching techniques with the MDAT. Figure 5(a) shows the XP profiles obtained with the EDR2 film in plastic water and those obtained with the CC04 Wellhofer ion chamber in water at depths of $d_{\max}$, 5 cm, 10 cm, and 15 cm for a $10\times 10\;{\text{cm}}^{2}$ field size. Measurements were also taken for the $5\times 5\;{\text{cm}}^{2}$ and $20\times 20\;{\text{cm}}^{2}$ field sizes, yielding similar results (data not shown). Figure 5(b) shows the PPDs obtained with the EDR2 film in plastic water and those obtained with the CC04 Wellhofer ion chamber in water for the $5\times 5\;{\text{cm}}^{2},10\times 10\;{\text{cm}}^{2}$, and $20\times 20\;{\text{cm}}^{2}$ field sizes. For both the XP profiles and the PDDs, the relative dose measurements showed that the results obtained with the film dosimetry system agreed within 3% in low‐dose gradient regions and within 2 mm distance‐to‐agreement (DTA) in high‐dose gradient regions (such as the buildup and penumbra regions) with the results obtained with the ion chamber. The slight differences observed in the PDDs of the $20\times 20\;{\text{cm}}^{2}$ field size are due to energy‐dependent variations in film sensitivity that were not accounted for in our SFC method. This observed effect, albeit small, is consistent with results previously reported in the literature.^(^ ^8^ ^–^ ^9^ ^)^

### B. MDAT

#### B.1 Dose bin size

In general, dose bin size in any histogram is selected based on the errors inherent in the experimental system.^(^ ^12^ ^)^ In our study, the error was determined from the reproducibility of the EDR2 film data. Based on the results obtained from the flat phantom film evaluation data, the largest variability for any technique (depth and field size combination) was on the order of 1.0%. In fact, as a whole, the average reproducibility was < 0.5% dose. Therefore, a dose bin size of 1% appeared to be reasonable and thus was used throughout the analysis in light of this maximum error.

**Figure 6:**
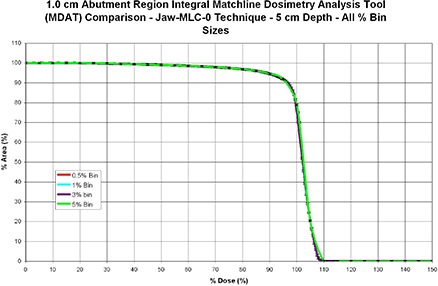
Cumulative DAHs of the Jaw–MLC–0° matching technique calculated using four dose bin sizes. Although the bin size is commonly defined by the inherent systematic error, we found that bin size has little effect on the shape of the DAH.

Although we suggest that the dose bin size be established on the basis of the film data's reproducibility, we used four dose bin sizes (0.5%, 1%, 3%, and 5% relative dose) to illustrate any differences between them. The integral MDAT for the Jaw–MLC–0° technique at a depth of 5 cm is shown in Fig. 6. With the possible exception of the 5% dose bin size, the shape of the integral MDAT was relatively unaffected by changes in the dose bin size. The MDATs for the other three dose bin sizes (0.5%, 1%, and 3%) are almost identical in shape. Thus, the highest resolution of the 0.5% dose bin size could have been used. However, since using dose bin sizes below the maximum stated error (~1%) could introduce artificial noise into the MDAT, we used the 1% dose bin size as the standard for comparison of the three matching techniques.

#### B.2 Abutment region width

**Figure 7:**
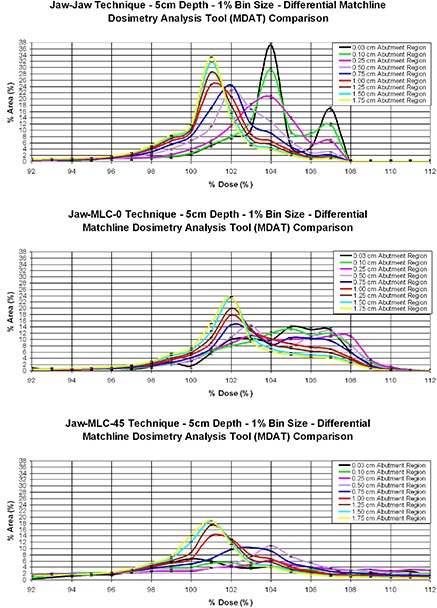
Abutment region widths for the three matching techniques. All graphs show a change in structure between the 0.75 cm and 1.0 cm abutment region widths. Regions wider than 1.0 cm appear to have a similar curve structure, whereas those narrower than 0.75 cm seem to illustrate a different curve structure.

To determine how the MDAT varies with abutment region width, we used the differential, rather than the integral, MDATs of all three matching techniques (Fig. 7). Of particular interest are the 0.50–1.00cm region widths, where the relative differential MDATs change shape.

One could argue that the 0.75 cm width would be a suitable selection for abutment region width because it possesses a shape that appears to “transition” between the smaller and larger abutment region widths. Conversely, the 1.0 cm width could also be a suitable selection because all widths greater than 1.0 cm display similar, if not the same, results. We selected the 1.0 cm abutment region width to display subsequent results of the various matching techniques throughout the remainder of this work. We did so primarily for one reason. While all flat phantom measurements were conducted with an MLC that has 0.5 cm leaf widths, our clinical breast measurements were conducted with the 1.0 cm leaves, due to the larger length of the fields. The 0.75 cm abutment region width is considered too narrow to evaluate a match produced by fields with wider leaf widths and with angled collimator settings.

Given the relative placement on the dose axis of the MDAT graphs, it was apparent that regardless of matching technique or abutment region width, the dose within the abutment region appears to be greater than the treatment‐field dose that was used for normalization. Figure 7 also shows that as the abutment region width becomes smaller (0.03–0.50cm), the average dose in the abutment region increases, suggesting a “hot” match. As the abutment region width becomes larger (1.00–1.75cm), the average dose in the region decreases, becoming closer to 100% relative dose. This occurs as a result of a larger proportion of the abutment region encompassing the portion of the field outside of the penumbra and, therefore, in the flat uniform dose region that was used to normalize the film.

### C. Evaluation of beam‐matching techniques using the MDAT: flat phantom

**Figure 8:**
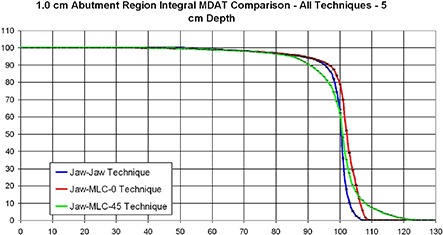
An integral dose‐area histogram (DAH) of the matchline dose analysis tool (MDAT) used to examine dose differences between the three beam‐matching techniques. Of particular interest are the shoulder and tail regions of the DAHs.

The results of analyzing the three beam‐matching techniques using the MDAT are shown in Fig. 8. As expected, the Jaw–Jaw technique produced the most uniform match, whereas the Jaw–MLC–45° technique produced the worst uniformity. The former is expected because of the appropriateness of the match geometry, and the latter because the Jaw–MLC–45° technique has a 45° collimator rotation that prevents the MLC leaves from forming a flush match. This MLC‐leaf geometry produces areas of 100% overlap or 100% exposure in the matchline region, thus creating the increased hot and cold regions seen in the shoulder and tail, respectively, of the graph of the integral MDATs.

### D. Evaluation of beam‐matching techniques using the MDAT: clinical breast fields

**Figure 9:**
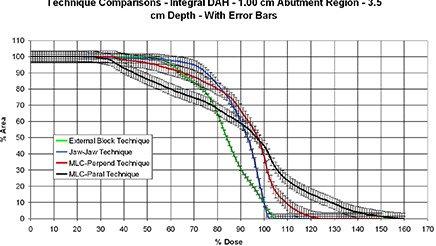
An integral dose‐area histogram (DAH) of the matchline dose analysis tool (MDAT) used to examine dose differences between the four clinical breast techniques.

A comparison of the four different tangential and supraclavicular beam matching techniques examined in the anthropomorphic phantom is shown in Fig. 9, to demonstrate dose homogeneity within the abutment region. Interpretation of the results of this figure is no longer as straightforward, since there is no one technique that appears to be superior to the others in all aspects. With regard to dose homogeneity, the flush matches (External Block and Jaw–Jaw) display the highest conformity, albeit still far inferior to the flat phantom results. Interestingly, it appears in all instances – except for the MLC‐Parallel technique – that the abutment region was cold relative to the tangent dose used for normalization. The external block technique, which was our standard technique before selection of an MLC‐defined match, appears to be colder than the other techniques, as well.

## IV. DISCUSSION

In this paper, we evaluate the distribution of dose existing at the junction of adjoining treatment fields. From our perspective, there is an interest in this topic, given the clinical situations in which such field geometries arise. Such a situation exists when treating the intact breast and associated lymphatics. Although IMRT may be thought of as a treatment modality that possibly mitigates the problem due to eliminating certain abutment field configurations all together,^(^ ^5^ ^–^ ^6^ ^)^ it is not universally used in all clinical situations or at all institutions where fields have historically been abutted.

Some questions to consider include the quantification of the degree to which dose inhomogeneities exist in a region of field abutment, and the degree to which these dose inhomogeneities can be improved. If the region of abutment is included in the plan's target volume, then is the resulting target DVH sensitive enough to demonstrate dose heterogeneity in the abutment region alone? We have chosen in this report to emphasize only the region of abutment where fields match. This region is carefully defined, and a tool is described which can be used to objectively characterize the dose distribution within it in a clinically meaningful way. The tool can then be used to sensitively describe the effects of field‐matching techniques on the resulting dose in the match region.

In the flat phantom portion of this work, we see that, from a dose uniformity perspective, the better field match is that produced using the Jaw–Jaw technique. Next, in terms of dose homogeneity, were the Jaw–MLC–0° technique and then the Jaw–MLC–45° technique. The Jaw–MLC–0° produces the second‐best match because the MLC leaves of the inferior field continue to form a flush match with the collimator jaw of the superior field. However, since inherent in the field blocking associated with this technique are rounded MLC leaves rather than a flat and divergent collimator edge, leaf transmission causes hot spots to form. One would expect the hot spot from this type of leakage to be higher than the hot spot in the Jaw–Jaw technique, which explains the results that are observed (~ 8%–9% hot spot for the Jaw–MLC–0° compared to ~ 6%–7% for the Jaw–Jaw). The Jaw–MLC–45° technique produces the least homogeneous dose distribution because of the collimator rotation. Due to the placement of the leaves relative to the matchline, certain areas in the abutment region will be either completely exposed or completely blocked, thus allowing portions of the abutment region to be either exposed for 100% of the dose from both fields or not exposed at all. This arrangement leads to hot spots in excess of 120%, and to roughly 10% of the match region receiving only 90% of the dose, as seen in Fig. 7.

Similarities exist between flat‐phantom and breast‐phantom results, from the perspective of dose homogeneity within the abutment regions. The dose within the matches formed with smooth appositional surfaces, such as with external blocks or collimator jaws, are more homogenous than the dose within matches formed with an angled MLC. In the breast phantom, we see that the magnitude of the nonuniformity is even more pronounced than in the flat phantom. There are some disadvantages associated with the two techniques that used the MLC to form the match in the breast phantom. For one, due to the increased length of the tangential fields, the 1.0 cm MLC leaves had to be used to form the match, rather than the 0.5 cm leaves that were used in flat phantom. Use of 1.0 cm leaves produces much larger areas either completely blocked or open, thus increasing the amount of inhomogeneity in the abutment region. The necessary use of 1.0 cm wide leaves for field matching was an important consideration when attempting to determine abutment region width. This point is considered in more detail later in this discussion.

The effect of collimator rotations on the dose uniformity in the match region was more significant in the breast phantom than it was in the flat phantom. The dose homogeneity throughout the entire treatment volume for the breast phantom, however, was similar regardless of the matching technique used. This fact caused us to reassess the way that we implement our breast‐tangent field‐in‐field dose modulation. The collimator rotation of the tangential fields is set anatomically, mostly to conform to the outline of the lung and block the lung with a collimator jaw. The leaves of the MLC are, in this orientation, perpendicular to the jaw blocking the lung. They produce a match along the SC field border represented by the MLC‐parallel configuration (Fig. 3(d)). This field orientation is also used for our field‐in‐field modulation. A more favorable match with the SC field is produced using MLC leaves that are perpendicular to the match line, the MLC‐perpendicular configuration (Fig. 3(c)). We have chosen to now use this second orientation, rather than the MLC‐parallel orientation, for the open‐field portion of the tangent field‐in‐field.

An important contribution of this work that may require highlighting is the suggested “width” of the abutment region. We expended significant effort in defining a meaningful width for this region. This was a necessary preliminary step before the MDAT could be used to compare beam‐matching techniques, particularly as fields defined by MLCs become the norm. The data of Fig. 7 suggest that the dose distributions in the region of the match should be evaluated within a width between 0.75 and 1.0 cm. We chose the larger 1.0 cm width, to accommodate fields defined by 1.0 cm thick leaves. For these leaves, the leaf half‐width would extend beyond the central matchline by 0.35 cm, assuming a 45° collimator rotation. The edges of the leaves would lie well within the boundaries of our 1.0 cm abutment region. This would not have been the case had we chosen the 0.75 cm abutment region width.

We also investigated the affect of daily setup on the MDAT. Compared to the results from the flat phantom, the error bars were much larger in the clinical breast studies. This reflects the much greater setup uncertainty associated with the measurements made in the anthropomorphic phantom. There were several additional steps required to set up the match plane for the clinical breast treatments. These included a couch kick to match the tangent and SC common border, and a table shift to allow for the two isocenter approach that we used: one for the tangents and one for the SC field. Treatment using a single isocenter technique, which would have limited the amount of shifting required, was not an option since doing so limits the available treatment field length.^(^ ^14^ ^)^ Additionally, while both the flat and breast phantoms had to be disassembled to conduct subsequent measurements, the clinical breast fields required a more intensive breakdown and setup as the entire phantom had to be completely removed from the table. Also, the breast phantom was placed on a breast board, setting it at an angle relative to the flat surface of the couch, rather than flush against the table, as was the case for the flat phantom measurements. Uncertainties in setup may, thus, conceal small dissimilarities in the dose distributions resulting from different field‐matching techniques.

Another difference that should be noted in the breast results is the “colder” DAHs for all of the matching techniques relative to the dose to which they are normalized. Recall that for the breast phantom measurements, only the dose within the tangent fields was used for normalization. It is assumed that the dose within the tangents equals the prescription dose. The dose of the SC field is prescribed to $d_{\max}$, resulting in a dose at the depth of abutment region analysis (3.5 cm) that is less than that within the tangents. Since half of the abutment region lies within the SC field, one would expect a cold match. The MDAT curves continue to demonstrate, however, dose relative to that which has been prescribed. Note also that relative dose homogeneity can still be examined, regardless of whether or not the two fields have the same dose at the measurement depth.

While the Jaw–Jaw match provides the most desirable abutment region homogeneity, it should be noted that the MLC must now be used to block the lung. This can lead to increased dose to the lung due to interleaf leakage (2% compared to ~ 0.5% with the collimator),^(^ ^15^ ^)^ but could be offset by the more conformal blocking of the lung by the MLC relative to a straight edge (i.e., collimator). Use of this configuration should be weighed against any possible gains in the matchline dosimetry when determining the appropriate treatment technique.

## V. CONCLUSIONS

We have showed that the MDAT, which is a quantitative tool that was developed to evaluate the dose distribution within the abutment region between two adjoining radiotherapy fields, is sensitive and descriptive enough to adequately assess the dose homogeneity in the abutment region of various field‐matching configurations and geometries, including clinical setups. Since the MDAT uses a graphical representation similar to the DVHs used for dose‐volume appraisal of target and normal‐structure volumes, its interpretation is very intuitive. In addition, it could also serve to determine mechanical uncertainties of collimator or leaf positions because of its sensitivity to determining hot and cold spots relative to the normalized field dose.

## ACKNOWLEDGEMENTS

This work was funded, in part, by a grant from Varian Medical Systems, Inc.
